# Statistical analysis plan of a randomized controlled trial to compare a restrictive strategy to usual care for the effectiveness of cholecystectomy (SECURE trial)

**DOI:** 10.1186/s13063-018-2989-5

**Published:** 2018-11-03

**Authors:** Sarah Z. Wennmacker, Aafke H. van Dijk, Joost P. H. Drenth, Sandra C. Donkervoort, Djamila Boerma, Gert P. Westert, Cornelis J. H. M. van Laarhoven, Marja A. Boermeester, Marcel G. W. Dijkgraaf, Philip R. de Reuver

**Affiliations:** 1 Department of Surgery, Radboud University Medical Centre, P.O. Box 9101, 6500 HB Nijmegen, The Netherlands; 2 Department of Surgery, Academic Medical Centre, Meibergdreef 9, 1105 AZ Amsterdam, The Netherlands; 3 Gastroenterology and Hepatology, Radboud University Medical Centre, P.O. Box 9101, 6500 HB Nijmegen, The Netherlands; 4 Department of Surgery, Onze Lieve Vrouwe Gasthuis, Oosterpark 9, 1091 AC Amsterdam, The Netherlands; 5 Department of Surgery, St. Antonius Hospital, Koekoekslaan 1, 3435 CM Nieuwegein, The Netherlands; 6 Department of IQ healthcare, Radboud University Medical Centre, P.O. Box 9101, 6500 HB Nijmegen, The Netherlands; 7 Clinical Research Unit, Academic Medical Centre, Meibergdreef 9, 1105 AZ Amsterdam, The Netherlands

**Keywords:** Cholecystectomy, Symptomatic gallstones, Abdominal pain, Cost-effectiveness

## Abstract

**Background:**

Cholecystectomy is the preferred treatment for symptomatic cholecystolithiasis. However, persistent pain after cholecystectomy for symptomatic cholecystolithiasis is reported in up to 40% of patients. The aim of the SECURE trial is to compare the effectiveness of usual care with a restrictive strategy using a standardized work-up with stepwise selection for cholecystectomy in patients with gallstones and abdominal complaints. The SECURE trial is designed as a multicenter, randomized, parallel-arm, non-inferiority trial in patients with abdominal symptoms and ultrasound-proven gallstones or sludge. Randomization was performed to either usual care (standard practice, according to the physician’s knowledge and experience, and physician’s and patient’s preference) or a restrictive standardized strategy: treated with interval evaluation and stepwise selection for laparoscopic cholecystectomy based on fulfilment of pre-specified criteria. This article presents in detail the statistical analysis plan (SAP) of this trial and was submitted before outcomes were available to the investigators.

**Results:**

The primary end point of this trial is defined as the proportion of patients being pain-free at 12 months’ follow-up. Pain will be assessed with the Izbicki Pain Score. Secondary endpoints will be the proportion of patients with complications due to gallstones or cholecystectomy, quality of life, the association between the patients’ symptoms and treatment, work performance, and cost-effectiveness.

**Discussion:**

The data from the SECURE trial will provide evidence whether or not a restrictive strategy in patients with symptomatic cholecystolithiasis is associated with similar patient reported outcomes and a reduction in the number of cholecystectomies compared to usual care. The data from this trial will be analyzed according to this pre-specified SAP.

**Trial registration:**

The Netherlands National Trial Register NTR4022. Registered on 5 June 2013.

## Introduction

Despite the high number of cholecystectomies for symptomatic cholecystolithiasis performed worldwide, this approach appears to be ineffective as up to 40% of patients will continue to have (persistent) abdominal pain [1, 2]. The SECURE trial is designed to examine the effectiveness of usual care versus restrictive strategy in patients with symptomatic gallstones. The trial protocol was previously published [3] and present article is the proposed statistical analysis plan (SAP). The SAP was written by the investigators before any of the outcomes were available.

## Summary study protocol

The SECURE trial (Scrutinizing (in)efficient use of cholecystectomy: a randomized trial concerning variation in practice) is designed as a multi-centre, randomized, parallel-arm, non-inferiority study in 1038 subjects with abdominal symptoms and ultrasound-proven gallstones or sludge.

All patients between the age of 18 and 95 who are referred to a surgical outpatient clinic for treatment of ultrasound-proven gallstones or sludge, and abdominal complaints are suitable for inclusion. Exclusion criteria are (a) a history of complicated cholelithiasis; (b) an indication for primary open cholecystectomy; (c) a history of current malignancy; (d) an expected short life span of less than 12 months; (e) an American Society of Anaesthesiologists physical status classification (ASA) of III and IV; (f) known liver cirrhosis; (g) cognitive disorders that predispose unreliable questionnaire responses; (h) insufficient knowledge of the Dutch language and (i) pregnancy.

After inclusion, patients will be randomized to either usual care or to the restrictive strategy. Patients assigned to the usual care group will receive the standard care given at the participating centres. Treatment decisions (i.e. cholecystectomy or conservative treatment) will be based on the physician’s knowledge, preference and experience, and on the patients’ preferred choice of treatment. The restrictive strategy includes a stepwise selection for surgery, using a triage instrument for symptomatic cholecystolithiasis. In this restrictive strategy, patients are selected for cholecystectomy on fulfilment of pre-specified criteria of the triage instrument: biliary colic defined by the Rome criteria, pain radiating to the back and a positive pain response to simple analgesics. Patients not fulfilling the pre-specified criteria are selected for conservative treatment and further work-up of alternative diagnoses for the abdominal complaints. Patients will be evaluated at the outpatient clinic every 3 months. Patients in the restrictive strategy with conservative treatment can be indicated for cholecystectomy at such an evaluation if the symptoms develop and match the pre-specified criteria, or if all other causes for abdominal pain are excluded.

The primary outcome is defined as the proportion of patients being pain-free at 12 months’ follow-up (irrespective of treatment). Pain and pain medication use will be assessed with the Izbicki Pain Score (IPS). Non-inferiority of the restrictive strategy to the usual care will be assessed, with a non-inferiority margin of 5% (i.e. significance level).

Secondary endpoints include number of cholecystectomies, time to pain-free, complications due to gallstones or cholecystectomy, patient-reported satisfaction on treatment outcome, alternative diagnostics and treatment, patients’ health status over time, working disability, the association between the patients’ symptoms and treatment, practice variation, and cost-effectiveness. Safety outcomes are complications of treatment, and serious adverse events (SAEs).

A total of 1038 evaluable patients should be included in this study. For power analysis, we assumed that the percentage of pain-free patients after 12 months in the restrictive strategy would be at least equal to the usual care, and that in the usual care a maximum of 80% of patients would be pain-free after 12 months. Restrictive strategy would be considered non-inferior if at least 75% of patients are pain-free. Possible contamination of the usual care by the restrictive strategy might increase the percentage of patients being pain-free (e.g. by 1%), and the non-inferiority boundary should increase equally. Therefore, with a one-sided Z-test, power of 80% and significance level of 5%, 1038 evaluable patients (519 per arm) need to be included. In absence of contamination this would result in a power of 89%. On 26 April 2017, all patients will be included and the completed follow-up is expected in May 2018. The power analysis and other study procedures are further detailed in the previously published trial protocol [3].

### Protocol developments

The SECURE trial is registered at the Netherlands National Trial Register (NTR4022) on 5 June 2013. The Institutional Review Board of the Academic Medical Centre, Amsterdam, approved the protocol on 7 August 2013, and 24 participating centres were added in the course of the study, located throughout The Netherlands.

## Statistical analysis plan

### General principles

The analyses will be performed after last patient out, after monitoring and cleaning of data gathered from all patients, and after acceptance of this SAP for publication. Analyses will be performed by the investigators and biostatistician of the SECURE study group, using the latest version of SPSS statistics (IBM Corp., Armonk, NY, USA).

### Patient flow diagram

**Fig. 1:**
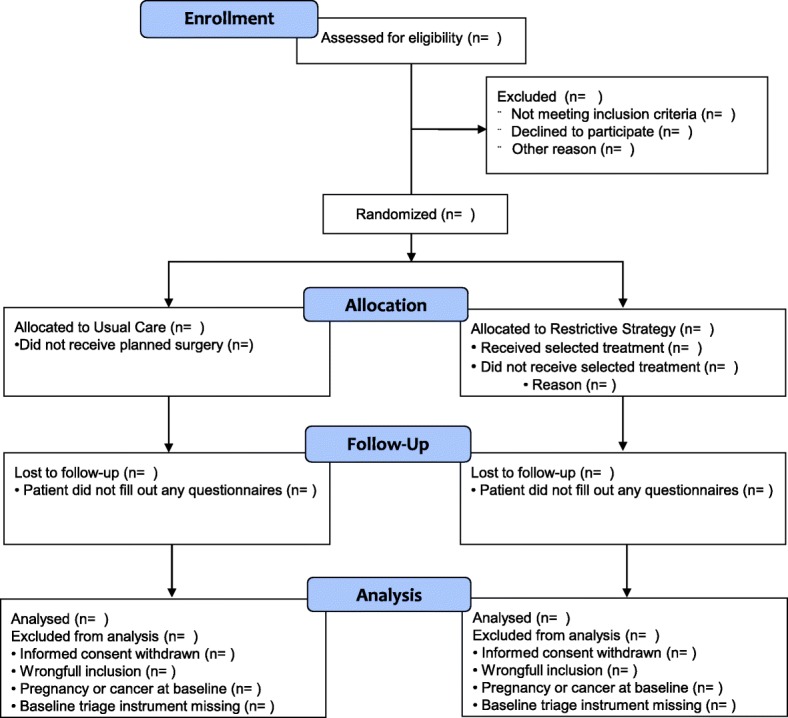
Participants flow diagram

The flow of participants will be illustrated in a flow diagram according the Consolidation Standard of Reporting Trials (CONSORT) (Fig. 1).

### Intention-to-treat and per protocol population

The main analysis will be performed according the intention-to-treat (ITT) principle. In the ITT analysis all patients are analysed according to their initially assigned study arm at baseline, regardless of adherence to study protocol. Patient who withdrew consent or patients with a protocol violation concerning eligibility are excluded from ITT analysis. Patients with missing baseline information on the fulfilment of the pre-specified criteria of the triage instrument and patients lost to follow-up (from whom no outcome data are available *at any time point* (baseline, 3, 6, 9, and 12 months follow-up)) were replaced and likewise excluded from ITT analysis. Differences in patient characteristics between patients lost to follow-up and included patients will be assessed. In case of potentially meaningful differences, a worst-case scenario will be run, in which all patients lost to follow-up in the usual care are considered pain-free, all patients lost to follow-up in the restrictive strategy are considered not pain-free after 12 months, and all patients lost-to-follow-up are added to the existing ITT analysis group. A full, reproducible account will be provided of what is or is not considered as potentially meaningful.

**Fig. 2:**
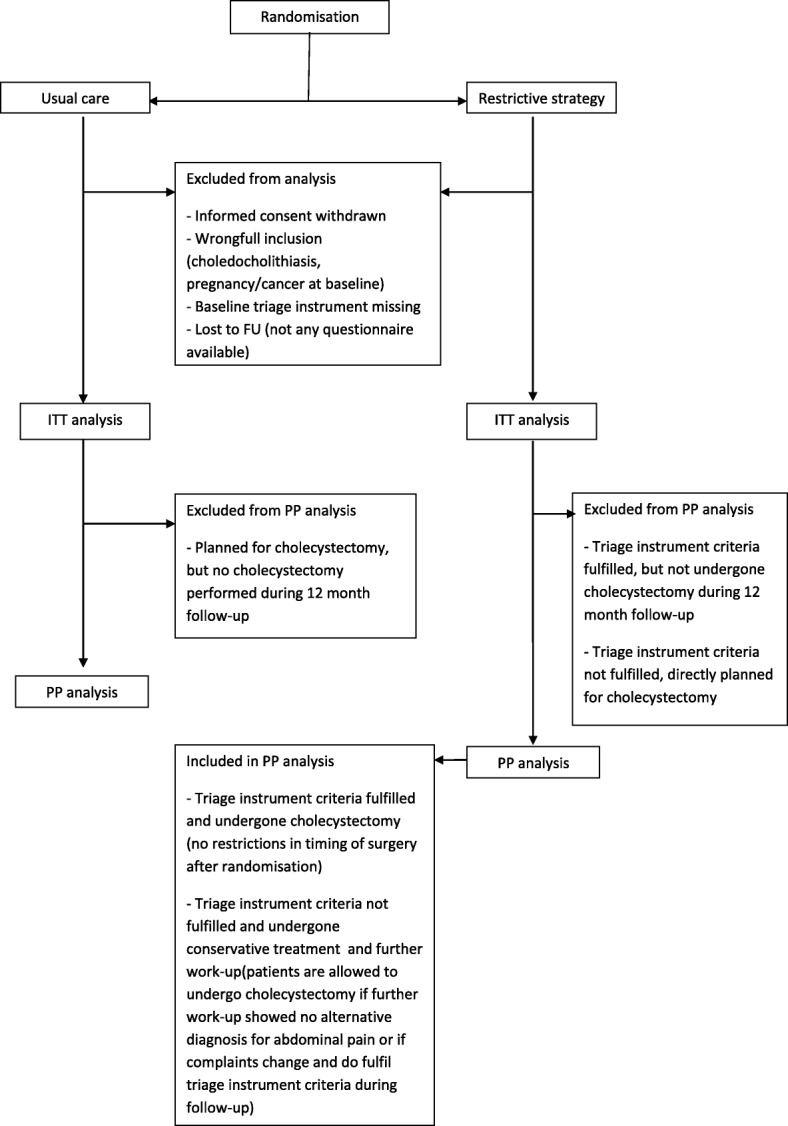
Patient inclusion for intention to treat (ITT) and per protocol (PP) analysis

In view of the non-inferiority study design, per protocol (PP) analysis will also be performed. All subjects from the ITT population without protocol violations and deviations regarding treatment will be included in the PP population (Fig. 2).

### Protocol violations in eligibility and consent, and deviations in treatment

Protocol violation in eligibility is defined as when a patient was randomized, but did no longer qualify for inclusion criteria or did meet an exclusion criterion (e.g. had choledocholithiasis or cholecystitis, was pregnant, had cancer at baseline). These patients will be excluded from further follow-up and analysis.

Informed consent is obtained from the patients according to the SECURE trial protocol [3]. Patients who withdrew their consent after randomisation will be excluded from further follow-up and analysis. The number of excluded patients after randomisation will be displayed in the CONSORT flow diagram (Fig. 1).

Patients assigned to the usual care arm (control) receive the standard care given at participating centres, based on physician’s knowledge, experience, and both, physician’s and patient’s preferences. Deviation of the usual care protocol is defined as when a patient is planned for cholecystectomy, but surgery is not performed during follow-up (Fig. 2).

Patients assigned to the restrictive strategy (intervention) are selected for cholecystectomy on fulfilment of the pre-specified criteria of the triage instrument, or selected for further work-up if patients did not meet the criteria of the triage instrument. The latter patients could undergo cholecystectomy during follow-up in the trial if abdominal symptoms developed and meet the criteria of the triage instrument at a later time point, or when further work-up shows no alternative diagnosis and gallstones are the only remaining explanation for the symptoms. Deviation of the restrictive strategy protocol is present if (1) patients who meet the criteria of the triage instrument for cholecystectomy do not undergo cholecystectomy, (2) patients who do not meet the criteria of the triage instrument do undergo early cholecystectomy, which does not refer to patients who undergo further work-up that shows no alternative diagnosis and gallstones are the only remaining explanation for the symptoms (Fig. 2).

### Patient replacement and missing data

Based on the sample size calculation we need to include a total of 1038 evaluable patient (519 per study arm). Patients are evaluable if they are not excluded due to protocol violation in eligibility or consent, and if (1) the triage instrument (with pre-specified criteria for cholecystectomy) at baseline is completed, and (2) primary outcome at 12 months (either directly or through imputation) is available. To reach the appropriate sample size and targeted power in the study, patients not fulfilling these evaluability criteria are replaced.

At the interim safety analysis in 2016, uncompleted baseline triage instruments and violations in patients’ eligibility and consent were observed. The expected rate of patients lost to follow-up and/or patients with incomplete data was 21%. The additional number of patients that need to be replaced is calculated according the formula 1038 / (1 - proportion non-evaluable patients). Based on this the total sample size was increased from 1038 to 1325 patients. Importantly, no changes were made regarding the primary outcome and secondary outcomes.

If outcome data at 12 months’ follow-up cannot be obtained through patients’ questionnaires, the patient will be contacted by telephone to retrieve the primary outcome. Secondary outcome data of these patients and all data on patients that cannot be contacted will be considered missing. Patients from whom no outcome data are available *at any time point* (baseline, 3, 6, 9, and 12 months follow-up) are considered lost to follow-up and will be excluded from analysis.

Different scenarios for handling missing data will be considered. A definitive choice for method of handling missing data will be made based on the robustness of the analysis results.

First, complete case analysis will be performed in which patients with incomplete data at 12 months’ follow-up will be excluded from analysis. Although the non-evaluable patients are replaced, this method could lead to loss of power and biased estimates [4]. Second, single imputation by last observation carried forward (LOCF) will be performed, using the results of the last available patients’ questionnaire (9, 6, 3 months’ follow-up or baseline). Third, the nearest neighbour (NN) imputation will be performed. The missing value will be replaced by a value obtained from related cases in the cohort, either the actual measured value from another patient (1-NN) or the average of measured values from several (k) patients (k-NN) [5]. Lastly, multiple imputation will be performed. The coefficients of multiple (% of missing data) rounds of imputation, based on relevant patient characteristics and outcome predictors, will be used to obtain final estimates of the missing value [6]. The variables selected as predictors for imputation contain; sex, age, body mass index (BMI), ASA classification, history of abdominal surgery, study arm, treatment, fulfilment of criteria of the triage instrument, complications, smoking, alcohol consumption, use of pain medication, not-pain-related abdominal complaints, and the questionnaires of all time points. The imputed values will be primarily estimated within the observed range of the IPS and visual analogue scale (VAS) pain score. In case the observed range is smaller than the total range (IPS 0–100 and VAS pain score 0–10 respectively), additional estimations within the total range of IPS and VAS pain score will be performed, to identify possible outliers.

The imputation strategy with the smallest confidence intervals and with point estimates closest to the complete case analysis results will be reported as the most robust one.

Missing values of baseline characteristics will not be imputed. When displaying the baseline characteristics, the actual denominator will be stated for dichotomous variables. For continuous variables a footnote to show the number of patients for whom the variable was available will be stated.

### Baseline characteristics

The baseline characteristics of the included patients will be reported per randomisation group and shown in a baseline table (Table 1). The following characteristics will be reported: age (years), sex (% F), BMI (kg/m^2^), ASA-classification (% ASA II), history of abdominal surgery (% yes), use of pain medication (% yes), type of medication (% paracetamol, nonsteroidal anti-inflammatory drugs [NSAIDs], other) and indication medication use (% used for gallstone symptoms), smoking (% yes, number of packs/week), alcohol consumption (% yes, number of glasses/week), baseline IPS, baseline VAS pain score, baseline health utility score (derived from the EuroQol 5 dimensions (EQ-5D) questionnaire) and baseline Gastrointestinal Quality of Life Index (GIQLI) score. Dichotomous variables will be summarised as proportion of patients with the count divided by the total number of evaluated patients. Continuous variables will be summarised as mean with standard deviation in case of normal distribution and as median with interquartile range in case of non-normal distribution. Testing for normality of data distributions will be based on the Shapiro-Wilks test. For continuous variables a footnote will state the number of evaluated patients. Differences in baseline characteristics between study arms will be reported as preferred by the intended journal. Differences between the study arms will then be analysed using chi-square test, independent *t* test or Mann-Whitney test if requested by the journal.

**Table 1:** Baseline characteristics

	Usual care (n)	Restrictive strategy (n)
Age, years		
Sex, F (% n/N)		
BMI, kg/m^2^		
ASA-classification, II (% n / N)		
History of abdominal surgery, yes (% n/N)		
Use of pain medication, yes (% n/N)		
Type of medication		
Paracetamol, n (% n/n)		
NSAID, n (% n/n)		
Other, n (% n/n)		
Indication of medication		
Gallstone symptoms, n (% n/n)		
Other indication, n (% n/n)		
Smoking, n (% n/N)		
Packs per week, n		
Alcohol consumption, n (% n/N)		
Glasses per week, n		
Baseline IPS, total points		
VAS pain score		
Baseline Health Utility score		
Baseline GIQLI-score, total points		

ASA American Society of Anaesthesiologists physical status classification, *BMI* body mass index, *GIQLI* Gastrointestinal Quality of Life Index, IPS Izbicki Pain Score, NSAID nonsteroidal anti-inflammatory drug, VAS visual analogue scale

### Assessment and analysis of primary outcome

The primary endpoint is defined as the proportion of patients being pain-free at 12 months’ follow-up. This endpoint is derived from the patient-reported IPS. Pain-free is defined as IPS ≤ 10 (with a VAS pain score ≤ 4). Non-inferiority will be established if the lower limit of the one-sided 95% confidence interval for the proportion of patients being pain-free at 12 months following restrictive strategy is within the absolute 5% margin below the proportion under usual care. The results of the ITT and the PP analysis should both allow such interpretation.

In a supplementary appendix, the analyses will be repeated with logistic regression to take into account that randomisation was stratified by center, sex, and body mass index. Non-inferiority will be established if the lower limit of the one-sided 95% confidence for the adjusted odds ratio of being pain-free at 12 months for patients under restrictive strategy relative to patients under usual care exceeds the critical odds ratio corresponding to the absolute 5% margin below the observed proportion under usual care.

### Assessment and analysis of secondary outcomes

The assessment and analysis of the secondary outcomes will be discussed separately for each secondary outcome. In all analyses, statistical uncertainties are expressed in 95% two-sided confidence intervals. A *p* value of < 0.05 will indicate statistical significance.

#### Number of cholecystectomies and number of patients being pain-free after cholecystectomy

The total number of cholecystectomies performed after 12 months in the two study arms will be reported. Whether a patient underwent surgery is registered by the physician in the CRF and verified by patients’ interview (by phone) and medical records at 12 month follow-up. The proportion of patients being pain-free (defined as for the primary outcome) in this subset of patients following cholecystectomy will also be reported per study arm. The chi-square test will be used to compare the outcomes between the study arms. These outcomes will be shown in a secondary outcome table (Table 2).

**Table 2:** Secondary outcomes

	Usual care (n)	Restrictive strategy (n)	*p*
Cholecystectomy, n (%n/N)			
Pain-free at 12 months, n (% n/n)			
Time to cholecystectomy in weeks, median (IQR)			
Conservative treatment, (%n/N)			
Pain-free at 12 months, (% n/n)			
Time to pain-free, median (IQR)			
Gallstone complications, n (%n/N)			
Choledocholithiasis, n (%n/N)			
Acute cholecystitis, n (%n/N)			
Biliary pancreatitis, n (%n/N)			
Cholangitis, n (%n/N)			
Colic with hospitalisation, n (%n/N)			
Gallstone complication preoperative, (% n/N)			
Surgical complications, n (%n/N)			
CDC I, n (%n/N)			
CDC II, n (%n/N)			
CDC III, n (%n/N)			
CDC IV, n (%n/N)			
CDC V, n (%n/N)			
Patient-reported satisfaction, NRS			
Working disability			
Absence, hours			
Loss of productivity, hours			
Non-trial related SAEs, n (%/N)			

*CDC* Claivien-Dindo classification, *IQR* interquartile range, *NRS* numerical rating scale, *SAE* serious adverse event

#### Time to pain-free

The moment the patient is free of pain, defined as IPS ≤ 10 (with a VAS score ≤ 4), can be deducted from the patients questionnaire. The IPS is measured at 3, 6, 9, and 12 months of follow-up. The first follow-up questionnaire the patient reports an IPS ≤ 10 (with a VAS score ≤ 4) will be considered the time to pain-free, providing that the patient remains pain-free during the remaining follow-up period. Considering the limited number of assessments over time, life tables will be generated for time to pain-free. The median times to being free of pain, the Wilcoxon Gehan statistic and the corresponding *p* value will be reported in the secondary outcome table (Table 2) with a graphical representation presented in a supplementary appendix.

#### Complications of gallstones and cholecystectomy

All complications of gallstones and complications of cholecystectomy will be reported per study arm. Complications should be reported by the physician to the trial coordinator at occurrence. Occurrence of complications will be verified with the patient at 3 months and 12 months’ follow-up, and with patient’s medical records at 12 months’ follow-up. Complications of gallstones are defined as occurrence of choledocholithiasis, acute cholecystitis, biliary pancreatitis, cholangitis, or a biliary colic that required hospitalisation. Although natural history of gallstones includes occurrence of complicated gallstone disease, the number of patients with complicated gallstone disease can differ based on the study arm and is therefore considered as complication in this study. Surgical complications are classified according to the Clavien-Dindo classification (CDC) [7]. Complications will be assessed on patient level (yes/no) and chi-square test will be used to compare the outcomes between the study arms. In the case > 25% of patients have multiple complications, assessment will be performed on complication level (n per patient) using Poisson-regression analysis. These outcomes will be shown in the secondary outcome table (Table 2).

#### Patient-reported satisfaction on treatment outcome

At 12 months’ follow-up, all patients were asked to express their satisfaction on the treatment outcome (either surgical or conservative) with a numerical rating scale (NRS) between 0 and 10 (0 being the worst and 10 being the best appraisal). The satisfaction on the treatment outcome will be reported, as mean with standard deviation if the data are normally distribution, and as median with interquartile range if the data are skewed. The independent *t* test or Mann-Whitney test will be used to compare the outcome between study arms. Testing for normality of data distributions will be based on the Shapiro-Wilks test. The outcome will be shown in the secondary outcome table (Table 2).

#### Alternative diagnostics and treatment

At 12 months’ follow-up, information on all diagnostic procedures and treatments related to abdominal pain were obtained, through patients’ interview (by phone) and medical records. For the conservatively treated patients (i.e. who did not undergo cholecystectomy), the proportion of patients that underwent alternative diagnostic procedures and/or treatments (including diagnosis) will be reported. The proportion of patients for whom an alternative diagnosis (other than gallstones) for the abdominal pain was determined, and type of diagnoses, will be reported.

#### Health status scores over time

**Fig. 3:**
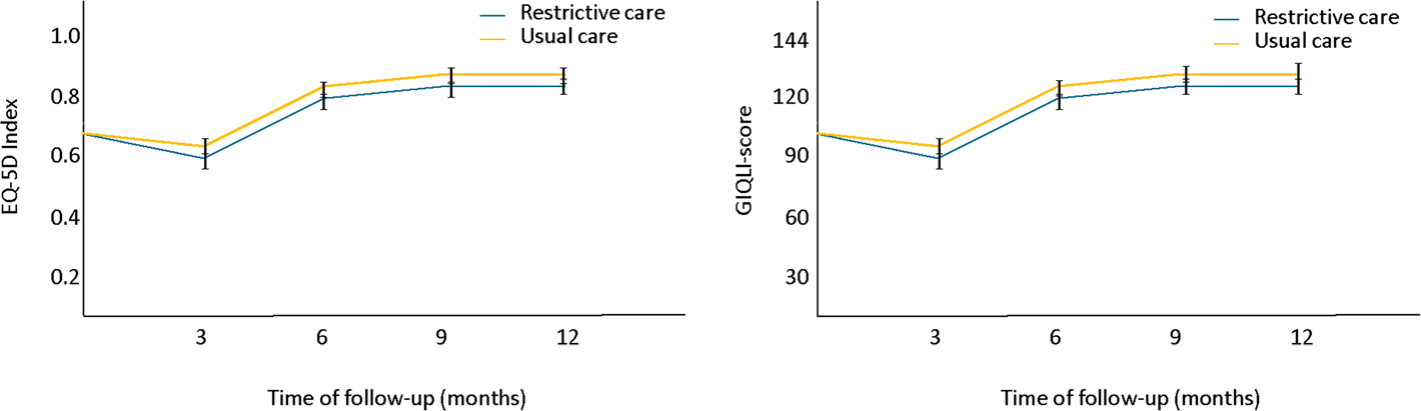
Example health status scores over time

Changes in disease-specific quality of life and health utility over time will be reported. Health status, defined by GIQLI scores, and health utility scores, derived from the EQ-5D-3 L profiles using the standard Dutch health valuation algorithm [8], is available at baseline and 3, 6, 9, and 12 months follow-up. The follow-up data will be assessed by repeated measurement analysis with baseline data as covariate, using a generalized linear mixed model. The results will be displayed per study arm in a graph (Fig. 3).

#### Working disability

Working disability will be assessed using the Short Form Health and Labour Questionnaire (SF-HLQ). Patients filled out this questionnaire at baseline, 3, 6, 9, and 12 months of follow-up, which provides information on absence from paid employment, production loss due to lower efficiency while at work and impediments to paid or unpaid employment. The cumulative number of hours of absence and production loss per study arm will be reported, as mean with standard deviation if the data are normally distribution, and as median with interquartile range if the data are skewed. The independent *t* test or Mann-Whitney test will be used to compare the outcome between study arms. Testing for normality of data distributions will be based on the Shapiro-Wilks test. (Table 2).

#### Cost-effectiveness and budget impact analysis

A cost-effectiveness analysis (CEA) with the costs per pain-free patient at 12 months as primary outcome measure will be performed. Additionally, a cost-utility analysis (CUA) will be performed with the costs per quality-adjusted life year (QALY) as outcome. The short- and mid-term affordability of the restrictive strategy in patients with abdominal pain and ultrasound-proven gallstones will be assessed from governmental, provider, and insurer perspectives following a budget impact analysis (BIA) [9]. The cost-effectiveness analyses and BIA will not be included in the primary article on the clinical study results and are not further elaborated here.

#### Practice variation

Practice variation in number of cholecystectomies in the usual care arm will be assessed amongst the participation hospitals. The practice variation indicator is defined as the number of patients with cholecystectomy on hospital level per 100,000 inhabitants in the catchment area of the hospital, and will be adjusted for relevant patient characteristics (i.e. age, sex, BMI). Results on practice variation will not be included in the primary article on the clinical study results and the statistical approach will not be elaborated here.

### Additional analysis

The preoperative symptoms of the included patients and fulfilment of the criteria of the triage instrument will be assessed and shown per study arm. The relation between specific patient characteristics or symptoms and (1) being pain-free at 12 months’ follow-up and (2) undergoing a cholecystectomy will be explored, to identify additional patient characteristics that are associated with being pain-free at 12 months follow-up and after cholecystectomy. The following characteristics and (sets of) symptoms will be assessed: sex, age, BMI, center (high versus low volume), ASA classification, history of surgery, use of pain medication (and type), baseline VAS pain score, baseline GIQLI score, baseline IPS, fulfilment of criteria of triage instrument (fulfilment of all criteria and subset of the criteria). The additional analyses will be performed using logistic regression with and without study arm as co-variable. Variables with a *p* value < 0.1 in univariable analyses will be included in multivariable logistic regression analysis and reported in a supplementary appendix. Subsequently, backward stepwise elimination will be used as further variable selection method. The results will be reported as adjusted odds ratio.

### Safety outcomes

Safety outcomes are all SAEs due to complications of gallstones or treatment and non-trial-related SAEs, between inclusion of the patient in the trial and 12 months of follow-up. Complications of gallstones and treatment are already incorporated in Table 2, as secondary outcome. The number of non-trial related SAE’s will be added in Table 2 and types of SAEs will be described. Statistical analysis to compare non-trial-related SAEs between study arms is as described for complications of gallstones and cholecystectomy.

### Authorship

The two PhD fellows (AD and SW) of the SECURE study group will share first authorship. The initiating and coordinating investigator (PR) will be second author and principle investigators MB and CvL will share senior authorship as second to last (MB) and last author (CvL). The other members of the SECURE study group will be co-authors. The local investigators who contributed to study design and data and manuscript discussion, and included at least 50 patients will also be co-authors, provided that there are no author number restrictions at the intended journal. Otherwise the local investigators with the highest inclusion rate and fulfilling the above criteria will be co-author. All other local investigators will be explicitly mentioned in the article as collaborators.

## Discussion

The aim of the SECURE trial is to compare the effectiveness and cost-effectiveness of usual care with a restrictive strategy using a pre-operative work-up with stepwise selection for cholecystectomy in patients with gallstones and abdominal complaints. The present article presents the analyses that will be published in the primary manuscript. This SAP was written to prepare for future analyses (before the data are available) and to increase transparency of scientific conduct, thereby allowing others to comment our plans.

Symptomatic gallstone disease is a very common condition with an estimated lifetime prevalence of 5–20% [10, 11]. Laparoscopic cholecystectomy is the treatment of choice to relieve symptoms, mainly abdominal pain. However, up to 40% of patients report persistent pain after surgery [1, 2]. Annually, approximately 24,000 laparoscopic cholecystectomies are performed in the Netherlands [12] and 700,000 in the USA [13]. The total estimated hospital-related costs of this procedure in the United States are USD 9.9 billion (based on 700,000 cholecystectomies and average hospital-related costs of USD 14,107 [14]). More than 60% of additional costs of employed patients are caused by costs related to sick leave of employees [15]. After a successful cholecystectomy time before return to work ranges from 1 to 10 weeks [16]. Consequently, the total costs related to this operation are many times higher than the billions of hospital-related costs per year. This means that performing a cholecystectomy without a realistic chance of a positive effect on complaints only adds to health care costs without health care gain. In addition, an unsuccessful cholecystectomy with persistent abdominal complaints adds an additional USD 555 per patient per year of medical costs and USD 361 per patient per year for sick leave from work and corresponding productivity loss [17]. These significant costs indicate the potential value of a more restrictive strategy for gallstone disease. An optimized indication for surgery could improve patients’ health status, prevent surgical complications, reduce health care demand and, consequently, lower health care costs. Therefore, we suggested a more restrictive strategy and hypothesized that the stepwise selection is non-inferior to usual care with respect to patient-reported outcome and could provide a basis to lower the number of cholecystectomies performed [3]. This might prevent further increase in cholecystectomy rates reported in the last decades and reduce the geographic variation between and within countries like France, Britain, the Netherlands and the USA [18]. The lack of level 1 evidence for the indication of gallbladder removal results in high operation rates and maintains practice variation.

Randomization in the SECURE trial was performed to either standard practice, according to the physician’s knowledge and experience and physician’s and patient’s preference (usual care), or a restrictive strategy: treated with interval evaluation and stepwise selection for laparoscopic cholecystectomy based on fulfilment of pre-specified criteria. A discussed pitfall of this study is the risk of contamination of usual care by the restrictive work-up approach. It is conceivable that specialists transfer their usual care to a more restrictive work-up during the trail. To reduce this potential bias the trial was performed in more than 20 Dutch hospitals and thereby many different surgeons were involved to avoid manifest and uniform contamination. Potentially remaining contamination was accounted for during the sample size calculation of the SECURE trial [3].

At interim safety analysis (after complete follow-up of 669 patients) violation of eligibility was noticed in 72 patients (10.8%) due to lack of baseline data on the triage instrument (not recorded by treating physician) and in 31 patients (4.6%) due to later discovered exclusion criteria (e.g. pregnancy or cancer). Additionally, 36 patients (5.4%) withdrew consent during follow-up and 6 patients (0.9%) were lost to follow-up. Due to this loss of evaluable patients the total number of included patients was increased from 1038 to 1325 patients to assure the availability of a total of 1038 evaluable patients for analysis of the primary endpoint.

The results of this trial may optimize the clinical outcome of gallbladder removal and reduce the number of ineffective operations. The Secure trial has the potential to impact daily clinical practice and improve patient outcomes. Second, a later cost-effectiveness and budget impact analysis of a more restrictive strategy may illustrate the cost reduction for health care providers, insurers, and society.

## Abbreviations

ASA: American Society of Anaesthesiologists physical status classification
BIA: Budget impact analysis
BMI: Body mass index
CDC: Claivien-Dindo classification
CEA: Cost-effectiveness analysis
CONSORT: Consolidation Standard of Reporting Trials
CUA: Cost-utility analysis
EQ-5D: EuroQol 5 dimensions
GIQLI: Gastrointestinal Quality of Life Index
IPS: Izbicki Pain Score
ITT: Intention-to-treat
LOCF: Last observation carried forward
NN: Nearest neighbour
NRS: Numerical rating scale
PP: Per protocol
QALY: Quality-adjusted life year
SAE: Serious adverse event
SAP: Statistical analysis plan
SF-HLQ: Short Form Health and Labour Questionnaire
VAS: Visual analogue scale
